# A Global Bibliometric Analysis of Publications on Recurrent Atrial Fibrillation After Radiofrequency Ablation

**DOI:** 10.7759/cureus.86396

**Published:** 2025-06-19

**Authors:** Mehmet Semih Belpinar

**Affiliations:** 1 Cardiology, Sivas Numune Hospital, Sivas, TUR

**Keywords:** atrial fibrillation recurrences, bibliometric analysis, publications, radiofrequency ablation, recurrent atrial fibrillation after radiofrequency ablation, visual analysis

## Abstract

Radiofrequency ablation for atrial fibrillation (AF) has become the standard therapy due to its high efficacy and recurrent complication potential. With the increased prevalence of cardiovascular diseases in an aging population, effective management remains a challenge. It is an area of active research and clinical emphasis, advancing ablation techniques that can provide new opportunities to optimize outcomes and minimize recurrence. The aim of this bibliometric investigation was to assess the publications of recurrent AF after radiofrequency ablation.

A thorough bibliometric analysis was performed using publicly available and subscription-based data obtained from the Web of Science database on the publications concerning recurrent AF following radiofrequency ablation. The present study focused on a qualitative analysis of publication volume trends and patterns in languages, affiliations, publishing houses, journals, countries, year-by-year publication, category, and co-occurrence of the major keywords.

The number of publications on recurrent AF after radiofrequency ablation has risen dramatically, with 276 studies involving 1763 researchers from 359 institutions in 35 countries. A total of 68.48% of papers are articles, while meeting abstracts contribute 25%, and review articles comprise 4% of the total. The number of publications has sharply increased in recent years, particularly in 2023 and 2024, with the highest number of publications being published in 2024. The average citations per document for the 10 most cited publications were 15.43. Important studies have provided essential insight into AF recurrence, while other studies have investigated predictors of AF recurrence, such as left atrial volume, as well as sleep apnea and corticosteroid therapy. China is the most voluminous contributor to the field, with 122 publications and 591 citations. Among the most remarkable countries, institutions, and journals, Japan and South Korea hold a significant share. Notably, Japan's University of Tsukuba stands out with a high citation count despite a relatively low number of publications. In total, the articles have been published across 96 journals, with the European Heart Journal contributing the highest number of publications. Topics such as ablation techniques, atrial fibrosis, and predictors of recurrence are increasingly prominent, reflecting the rapidly evolving landscape of this research domain.

There has been a dramatic increase in relevant publications, with China at the center of production and cooperation. Landmark studies have significantly improved our understanding of recurrence predictors and treatment outcomes. New trends in ablative techniques, as well as factors surrounding recurrence, continue to highlight progression within the field.

## Introduction and background

Atrial fibrillation (AF) is one of the most common clinical arrhythmias; epidemiologic studies indicate that approximately 2% of the world's population suffers from AF [1].

Percutaneous radiofrequency catheter in medically refractory AF, which does not respond well to medications, can be treated with ablation. With this technique, the arrhythmogenic focus is electrically disconnected from the left atrium by means of catheters placed in the left atrium [2,3]. Radiofrequency ablation is a procedure to treat AF by burning targeted heart tissue. During the electrophysiologic study, accessory pathways and critical areas that cause AF are identified. Once the target area is identified, a catheter is used to heat this area above 47°C to cause cell death.

In recent years, with a better understanding of the mechanisms causing AF, ablation techniques have been developed, and this method is increasingly used in the treatment of AF [2]. Radiofrequency ablation, one of the most effective methods to control AF rhythm and maintain sinus rhythm, is strongly recommended as the first-line treatment in many guidelines [3]. "Recurrence of AF after radiofrequency ablation" refers to the recurrence of AF after radiofrequency ablation. In other words, it refers to the recurrence of arrhythmia after radiofrequency ablation for the treatment of AF. Recurrence of AF after radiofrequency ablation is observed within three months in 50% of patients. Early and multiple recurrences increase the risk of late recurrences within one year, which occur in 20-50% of patients [4]. Early recurrences after AF ablation are common in clinical practice. Observational studies have measured early recurrences using so-called "gap periods" ranging from 48 hours to three months after ablation. These studies reported early recurrence in 25-65% of patients undergoing AF ablation [5]. Late AF recurrence occurs between three months and one year after ablation, with a rate of 20-50%. Predictors of a late recurrence include age, hypertension, structural heart disease, and nonparoxysmal AF [4].

While there is no clear consensus on patient selection and timing for re-ablation, symptomatic patients with multiple recurrences and persistent AF are referred for ablation [4]. A recent study aimed to assess the risk of AF recurrences at six and 12 months after a single radiofrequency catheter ablation in patients with paroxysmal AF, including 40 patients. In this study, electrocardiogram (ECG) monitoring was performed at one, three, six, and 12 months, and participants monitored their own cardiac activity. In the study, a six-month risk score was created using three independent predictors: the duration of atrial fibrillation rate (AFR) in the first month, the number of AFRs between one and three months, and the number of supraventricular ectopic beats at six months. This score explained 59% of the AFR. At the 12-month assessment, the presence of AFR between six and 12 months was found to be the most important predictor, accounting for 45% of AFR. Summing up, the six-month score was helpful in guiding treatment strategies for low-risk patients, and long-term follow-up may prevent unnecessary early interventions [6]. The basic strategy of re-ablation is re-isolation of reconnecting pulmonary veins and ablation of non-pulmonary vein triggers. In addition to re-ablation, weight loss, treatment of sleep disturbances, and management of comorbid conditions are recommended to achieve and maintain a permanent sinus rhythm [4].

Bibliometric analysis is a method that uses scientific data to identify research trends, their impacts, and important authors. It reveals developments in the research field by examining data, such as publication numbers, citations, and impact factors. It also analyzes collaborations between authors and institutions [7-9]. Although there are bibliometric analyses in many medical fields, including cardiology, there is no similar publication on "recurrent AF after radiofrequency ablation" in the literature. Due to the existing deficiency, this study was designed to assist scientific researchers in this field.

## Review

Materials and methods

Search Strategy

A comprehensive bibliometric analysis of research papers on recurrent AF after radiofrequency ablation was conducted in this study. This cross-sectional study was carried out using publicly accessible and subscription-based data from the bibliographic database Web of Science (WOS).

Study Design

The literature was searched from its inception to March 7, 2025. Literature search and data downloads were done on a single day (March 7, 2025) to minimize bias arising from database updates. A comprehensive search on the WOS database (Clarivate Analytics, Philadelphia, USA) was conducted using the following search strategy: (Title) AND recurrent AND radiofrequency AND atrial fibrillation OR atrial fibrillation recurrences AND radiofrequency (Title). A comprehensive overview of publication trends and patterns has been provided, encompassing all types of documents published to date. Only English-language publications were included.

Data Collection

The WOS file was stored in txt format. Language, affiliations, publishers, journals, countries from where authors published their articles, year-by-year publication, category or general subject in which research was published, and co-occurrence of significant keywords in the published literature were all included in the data.

In the initial search, 296 publications were identified based on the keywords. A total of 19 publications were excluded due to their irrelevance to the topic, and one article that was not in English was also excluded. The remaining 276 publications were included in the study, and further analyses were conducted on these publications. Table 1 outlines the process of selecting relevant publications for the study.

**Table 1:** The process of selecting relevant publications for the study

Step	Action	Result
1. Initial search	Conducted search using keywords	296 publications identified
2. Exclusion of irrelevant publications	Exclude publications irrelevant to the topic	19 publications excluded
3. Exclusion of non-English articles	Exclude non-English publications	1 publication excluded
4. Remaining publications	Include remaining publications in the study	276 publications included
5. Further analysis	Apply further analysis to the remaining publications	Further analyses conducted on 276 publications

Data Analysis

Data were shown as numbers and percentages. As we presented the data in figures and tables, we noted the numbers used to compute the percentages. Graphical representations were created using Microsoft Excel 2010 (Microsoft® Corp., Redmond, WA, USA) and VOSviewer version 1.6.19 (Centre for Science and Technology Studies, Leiden University, the Netherlands) [10] to clearly present the results.

Results

There were 276 publications in total. These publications were written by 1,763 authors from 359 different institutions across 35 different countries. The distribution of publications by document type was as follows: 189 articles (68.47%), 69 meeting abstracts (25.00%), and 11 review articles (3.98%). The remaining 12 documents (4.34%) were categorized as "other." The publications were categorized according to the WOS index as follows: 268 in the Science Citation Index Expanded (SCI-EXPANDED), 46 in the Conference Proceedings Citation Index - Science (CPCI-S), eight in the Emerging Sources Citation Index (ESCI), and three in the Social Sciences Citation Index (SSCI). Some publications appeared in several categories. The publications were categorized based on their Open Access characteristics as follows: 144 in the All Open Access category (52.17%), 94 in the Gold category (34.05%), nine in the Gold-Hybrid category (3.26%), 27 in the Free to Read category (9.78%), 77 in the Green Published category (28.04%), two in the Green Accepted category (0.73%), and 19 in the Green Submitted category (6.91%). However, due to the fact that some publications fell into more than one category, the totals do not sum to 276. This can be attributed to the fact that certain publications fall under multiple open-access types.

Distribution of Publications and Citations by Years

**Figure 1:**
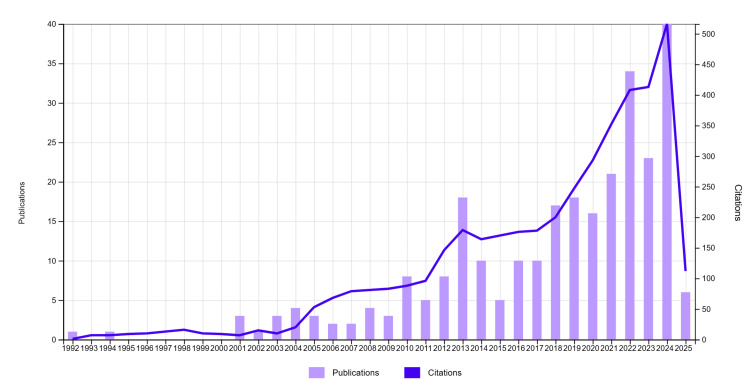
Number of publications and citations by years This figure shows the yearly trend in academic output (publications) and their impact (citations). The purple bar represents the volume of published works, while the blue line indicates the frequency of their citations, helping to assess research growth and influence over time.

The first publication appeared in 1992 [11]. The number of scientific publications has increased over the years, reaching its maximum in 2024 at 40 (14.49%). This trend is notably focused on the years 2024 and 2023, where the number of publications is the highest, as also in 2024 and 2023, with 63 (22.82%) publications. In previous years, there were few publications, with only five publications in 2015 and eight publications in 2010 and 2012. On the other hand, in recent years, a sharp rise occurred, with 63 publications (22.82%) in 2023 and 2024, and a minimum level in 2025, with only six publications (2.17%). In 2024, the highest peak of 40 publications (14.49%) indicates that the near future will see a rapid pace of scientific studies. The strong trend of scientific publication continues with 23 publications (8.33%) in 2023. The publication activity is mainly focused on 2024 and 2023. Specifically, an increase in the number of citations along with time was observed in the preceding five years. In 2024, it received 516 citations along with the highest number of publications. Figure 1 illustrates the trend over the years.

Most Cited Documents

Recurrent AF after radiofrequency ablation has been cited 4259 times with an average of 15.43 citations per item. The h-index of those publications is 29. The top three most cited articles reflect substantial contributions to our understanding of the impact of radiofrequency ablation on AF treatment. The article that received the most citations from 2005 was Hindrick et al.'s [12] "Perception of atrial fibrillation before and after radiofrequency catheter ablation - Relevance of asymptomatic arrhythmia recurrence" (source: Circulation, n = 364). In our current era of AF management, where much of our treatment targets PV ablations, Cappato et al. [13] highlight the study carried out by Hu et al. in 2003, titled "Prospective assessment of late conduction recurrence across radiofrequency lesions producing electrical disconnection at the pulmonary vein ostium in patients with atrial fibrillation," which was also published in Circulation and has received 336 citations; this study provides significant insight into the quality of treatment for AF through the re-establishment of electrical connections across the pulmonary vein ostium. Additionally, Njoku et al.'s 2018 meta-analysis [14], "Left atrial volume predicts atrial fibrillation recurrence after radiofrequency ablation: a meta-analysis" (source: Europace, 272 citations), is about a very important predictor for AF recurrence after radiofrequency ablation: left atrial volume. The remaining articles that report high citation rates highlight important variables impacting the recurrence of this treatment, including sleep apnea, corticosteroid therapy, and electrophysiological monitoring. Table 2 displays the most cited articles on radiofrequency ablation in AF, highlighting key findings and the factors influencing recurrence.

**Table 2:** Most cited documents

Title	Authors	Source Title	Publication Year	DOI	Total Citations	Average per Year
Perception of atrial fibrillation before and after radiofrequency catheter ablation - relevance of asymptomatic arrhythmia recurrence	Hindrick, et al. [12]	Circulation	2005	10.1161/CIRCULATIONAHA.104.518837	364	17.33
Prospective assessment of late conduction recurrence across radiofrequency lesions producing electrical disconnection at the pulmonary vein ostium in patients with atrial fibrillation	Cappato, et al. [13]	Circulation	2003	10.1161/01.CIR.0000091081.19465.F1	336	14.61
Left atrial volume predicts atrial fibrillation recurrence after radiofrequency ablation: a meta-analysis	Njoku, et al. [14]	Europace	2018	10.1093/europace/eux013	272	34
Predictors of atrial fibrillation recurrence after radiofrequency catheter ablation: a systematic review	Balk, et al. [15]	Journal of Cardiovascular Electrophysiology	2010	10.1111/j.1540-8167.2010.01798.x	196	12.25
Concomitant obstructive sleep apnea increases the recurrence of atrial fibrillation following radiofrequency catheter ablation of atrial fibrillation: clinical impact of continuous positive airway pressure therapy	Naruse, et al. [16]	Heart Rhythm	2013	10.1016/j.hrthm.2012.11.015	170	13.08
Improvement in left-ventricular systolic function after successful radiofrequency his-bundle ablation for drug refractory, chronic atrial fibrillation and recurrent atrial-flutter	HEINZ, et al. [11]	American Journal of Cardiology	1992	10.1016/0002-9149(92)90991-7	140	4.12
Repeat ablation for atrial fibrillation recurrence post cryoballoon or radiofrequency ablation in the Fire and Ice Trial	Kuck, et al. [17]	Circulation: Arrhythmia and Electrophysiology	2019	10.1161/CIRCEP.119.007247	126	18
Prevention of atrial fibrillation recurrence with corticosteroids after radiofrequency catheter ablation a randomized controlled trial	Koyama, et al. [18]	Journal of the American College of Cardiology	2010	10.1016/j.jacc.2010.04.057	125	7.81
Role of transtelephonic electrocardiographic monitoring in detecting short-term arrhythmia recurrences after radiofrequency ablation in patients with atrial fibrillation	Senatore, et al. [19]	Journal of the American College of Cardiology	2005	10.1016/j.jacc.2004.11.050	123	5.86
Use of contact force sensing technology during radiofrequency ablation reduces recurrence of atrial fibrillation: a systematic review and meta-analysis	Afzal, et al. [20]	Heart Rhythm	2015	10.1016/j.hrthm.2015.06.026	104	9.45
Predictors of recurrence following radiofrequency ablation for persistent atrial fibrillation	McCready, et al. [21]	Europace	2011	10.1093/europace/euq434	101	6.73
Cryoballoon versus radiofrequency catheter ablation of paroxysmal atrial fibrillation: biomarkers of myocardial injury, recurrence rates, and pulmonary vein reconnection patterns	Kuehne, et al. [22]	Heart Rhythm	2010	10.1016/j.hrthm.2010.08.028	101	6.31
Early recurrence of atrial tachyarrhythmias following radiofrequency catheter ablation of atrial fibrillation	Andrade, et al. [23]	Pace-Pacing and Clinical Electrophysiology	2012	10.1111/j.1540-8159.2011.03256.x	98	7
Prevalence of asymptomatic recurrences of atrial fibrillation after successful radiofrequency catheter ablation	Oral, et al. [24]	Journal of Cardiovascular Electrophysiology	2004	10.1046/j.1540-8167.2004.04055.x	95	4.32
Prognostic value of total atrial conduction time estimated with tissue Doppler imaging to predict the recurrence of atrial fibrillation after radiofrequency catheter ablation	den Uijl, et al. [25]	Europace	2011	10.1093/europace/eur186	84	5.6

Number of Publications and Citations by Years

**Figure 2:**
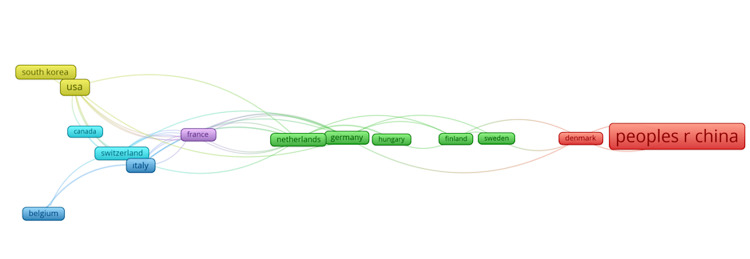
The co-authorship between countries The node size represents the number of publications from a country. Line thickness indicates the strength of collaboration between countries. Proximity and colors denote different countries or clusters of closely collaborating nations. This network visualization highlights international research collaborations. Countries with stronger connections (thicker lines) collaborate more frequently, while larger nodes denote higher publication output. Peoples r china: People's Republic of China (PRC) Image Credits: Mehmet Semih Belpinar

The top countries in terms of research productivity and citation impact are led by the People's Republic of China, with 122 publications and 591 citations, followed by Japan with 38 publications and 564 citations. The USA ranks third in publications (25) but leads in citations with 993, despite having fewer publications (25) compared to China. Italy had a strong citation impact with 604 citations from 10 publications, while Japan produced 38 publications and received 564 citations. South Korea contributed 13 publications and garnered 229 citations. China stands as the most prolific contributor, while the USA dominates with the highest citation count (Table 3). The co-authorship between countries is illustrated in Figure 2, which visually represents the collaboration patterns across different nations. This figure highlights the networks of co-authorship, showcasing the strength of research ties between countries. Countries with higher publication counts, such as China, the USA, and Japan, are likely to be central nodes in this network, collaborating extensively with other nations. The figure visually highlights how these countries collaborate, forming a global network of research cooperation and knowledge exchange.

**Table 3:** Distribution of documents, citations, and link strength by country

Country	Number of Documents	Number of Citations	Total Link Strength
People's Republic of China	122	591	2890
Japan	38	564	1233
USA	25	993	994
South Korea	13	229	606
Italy	10	604	556
Belgium	9	53	700
Netherlands	9	327	629
Switzerland	8	249	382
Germany	7	591	451
Poland	6	37	190
Denmark	4	39	256
England	4	190	288
France	4	126	195
Turkey	5	64	152
Canada	3	118	332
Romania	3	18	344
Russia	3	0	84
Spain	3	127	279
Taiwan	3	46	213

**Figure 3:**
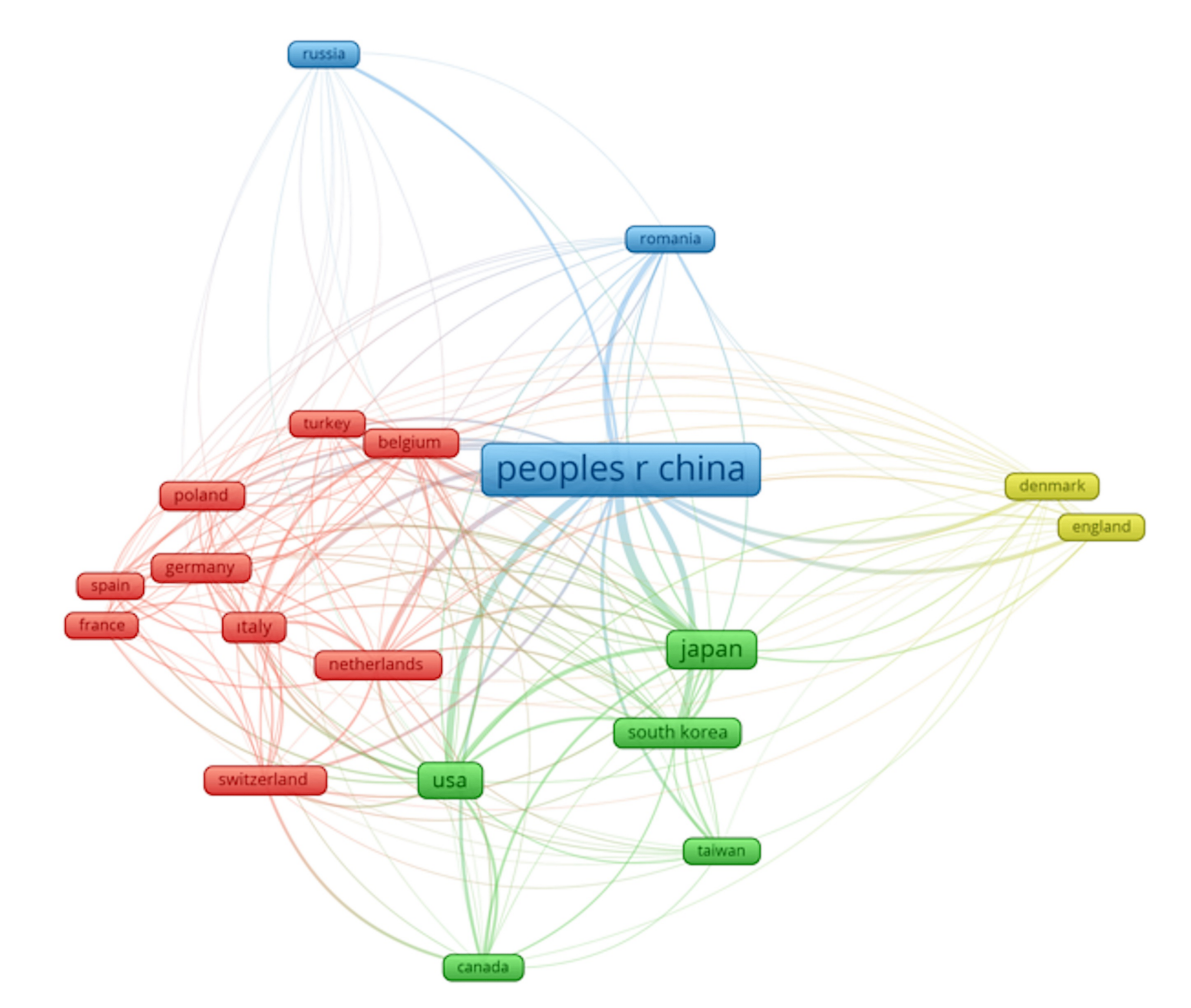
The bibliographic coupling between countries The node size reflects the total number of shared references. Line thickness shows the strength of similarity in research focus. Proximity and colors denote groups of countries with similar research themes. Bibliographic coupling measures the frequency with which countries cite the same sources, indicating thematic similarities. Closer and thicker links suggest overlapping research interests. Peoples r china: People's Republic of China (PRC) Image Credits: Mehmet Semih Belpinar

Figure 3 illustrates bibliographic coupling between countries, with countries that are closely connected represented in the same colors. The thickness of the lines increases as the level of connection between countries grows, indicating stronger bibliographic coupling. Countries sharing more common references in their publications are shown with thicker lines, highlighting the extent of their research alignment and collaboration. This visualization provides a clear view of how countries' scientific efforts overlap and the strength of their interconnectedness in terms of shared academic references. The total link strength reflects the level of global interconnectedness and collaboration within a country's research output. China leads with a total link strength of 2890, indicating its research is highly integrated into the global scientific community, followed by Japan with 1233, showcasing its strong international ties despite fewer publications. The USA has a total link strength of 994, highlighting its significant influence and widespread research connections. Countries such as South Korea (606) and Italy (556) also demonstrate strong collaborations, while Belgium (700) and the Netherlands (629) show considerable international research connections. In contrast, countries such as Russia (84) and Romania (344) exhibit lower total link strengths, suggesting more limited global collaboration. Overall, the data highlights the dominance of China and Japan in international research networks (Table 3).

The Most Productive Countries and International Collaborations

The People's Republic of China leads in research productivity and citation impact, producing 122 publications that have received 591 citations, followed by Japan with 38 publications and 564 citations. The USA ranked third by publication number (25) and estimated field citation (993). Despite a lower volume of publications (25) compared to China, Italy had a notable citation impact, with 604 citations across 10 publications. Japan, in contrast, published 38 publications and received 564 citations. A total of 13 publications from South Korea received 229 citations. China is the largest contributor, but the USA is the most frequently cited (Table 3). Table 3 presents the co-authorship of countries, with data types displayed and visualized. This figure illustrates networks of co-authorship, exposing the robustness of research connections among countries. Countries where most publications originate (such as China, the USA, and Japan) will probably be key nodes in this network, having collaborated with both countries with lower outputs and other larger nodes. This figure easily illustrates how these countries interleave with each other, forming a web of global research cooperation and knowledge exchange.

Figure 3 shows bibliographic coupling across countries, highlighting those that cluster together in the same colors. An increasingly thick line symbolizes a strong level of connection between countries with a higher degree of bibliographic coupling. Countries with greater co-citations in their publications are connected by thicker lines, demonstrating relevance and potential cooperation among their respective research sections. This visualization provides a clear view of the overlap in countries' scientific efforts and the degree of connection between them through shared references in academia. The total link strength indicates the amount of global interdependence and international cooperation in a country's research output. China ranks first, with a link strength of 2,890, indicating that its research is deeply embedded within the global scientific community. Japan comes second, with a link strength of 1,233, indicating that although the country publishes less, its research is well-established internationally. With a total link strength of 994, the USA is the strongest country in terms of link strength and arguably has the widest reach for research. South Korea (606) and Italy (556) are also well-connected, and Belgium (700) and the Netherlands (629) exhibit significant internal research links. Conversely, countries with low total link strengths, such as Russia (84) and Romania (344), are indicative of more isolationist sights. Overall, the data reveal that China and Japan dominate the global research networks (Table 3).

The Most Productive Institutions

Among the 359 institutions, 35 institutions published at least three articles. Table 4 provides an overview of the research impact by affiliation, showing the number of documents, citations, and total link strength for various institutions across different countries.

**Table 4:** Research impact by affiliation

Affiliation	Country	Number of Documents	Number of Citations	Total Link Strength
Capital Medical University	China	19	90	857
Soochow University	China	12	65	529
Dalian Medical University	China	11	73	376
Nanjing Medical University	China	7	31	437
Zhejiang University	China	7	43	305
Korea University	South Korea	6	113	198
University of Tsukuba	Japan	6	328	131
China-Japan Friendship Hospital	China	5	32	389
Chinese Academy of Medical Sciences & Peking Union Medical College	China	5	27	342
Kyoto University	Japan	5	3	192
Shanghai Jiao Tong University	China	5	54	170
Xuzhou Medical University	China	5	13	360
Zhengzhou University	China	5	18	347
Chinese People's Liberation Army General Hospital	China	4	12	167
Hebei Medical University	China	4	21	90
Leiden University	Netherlands	4	142	154
Nanchang University	China	4	15	188
Sakurabashi Watanabe Hospital	Japan	4	35	233
Tenri Hospital	Japan	4	3	184
University of Michigan	USA	4	99	42
Chang Gung University	Taiwan	3	46	144
Chinese Academy of Medical Sciences	China	3	23	151
Fujian Medical University	China	3	3	32
Fukushima Medical University	Japan	3	50	96
Gunma Prefectural Cardiovascular Center	Japan	3	17	59
Hyogo College of Medicine	Japan	3	3	184
Nanjing University	China	3	22	127
Ogaki Municipal Hospital	Japan	3	3	184
Okayama Heart Clinic	Japan	3	3	184
Southwest Jiaotong University	China	3	14	158
Université libre de Bruxelles (VUB)	Belgium	3	18	111
Vrije Universiteit Brussel	Belgium	3	9	70
Wenzhou Medical University	China	3	20	159
Yonsei University Health System	South Korea	3	96	146
Isala Klinieken	Netherlands	3	174	42

Capital Medical University in China has the highest number of documents and link strength in the global research network, followed by Soochow University, Dalian Medical University, and Nanjing Medical University. These institutions demonstrate China's dominance in research volume and interconnectedness. South Korean Korea University and Japan's University of Tsukuba also contribute significantly, with Korea University contributing six documents and 113 citations, and the University of Tsukuba having 328 citations despite publishing fewer documents. Japanese institutions such as Sakurabashi Watanabe Hospital and Kyoto University contribute to the global research network, exhibiting diverse citation counts and link strengths (Table 3).

Table 3 shows that Chinese institutions dominate in terms of publication numbers and total link strength, while institutions from Japan, South Korea, the Netherlands, and Belgium also contribute to the research landscape, but with lower publication volumes. The total link strength across institutions reflects the extent of their research collaborations and global influence, with China leading the way in both the number of publications and international research connections. Institutions like Leiden University, Université libre de Bruxelles, and Isala Klinieken have moderate citation counts and link strengths.

**Figure 4:**
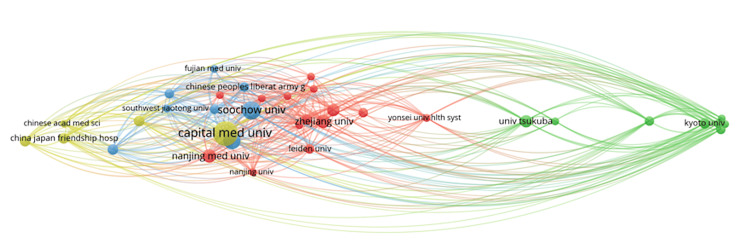
The bibliographic coupling between affiliations The node size represents the institution's contribution based on shared references. The lines indicate research similarity between institutions. Proximity and colors denote clusters of institutions with aligned research topics. This figure illustrates the thematic connections between research institutions based on their research similarity patterns. Stronger links imply similar academic focus areas. Image Credits: Mehmet Semih Belpinar

Figure 4 shows research impact and affiliations using VOSviewer, highlighting connections between institutions based on co-authorship and citation relationships. Chinese institutions, such as Capital Medical University and Soochow University, form a central cluster, demonstrating high publication rates and interconnectedness within the global research network. Institutions from Japan, South Korea, and the Netherlands are positioned separately, demonstrating limited but notable international collaboration. China is a clear focal point in the network.

Journal Analysis, Co-cited Journal Analysis

Publications on the topic of "recurrent AF after radiofrequency ablation" have been published in 96 different journals. Table 5 lists the top publishing journals in this field, with the European Heart Journal leading in volume but having a lower citation count. Circulation, with a smaller publication count but an impressive 706 citations, has a strong influence and high impact. Journals such as the Journal of the American College of Cardiology and Europace strike a balance between consistent publication and high citation rates, underscoring their importance in cardiovascular research. Other journals, such as BMC Cardiovascular Disorders and PACE: Pacing and Clinical Electrophysiology, have slightly lower citation counts, suggesting a niche focus or emerging influence. Journals like the Journal of Cardiothoracic Surgery and Heart show minimal citation impact.

**Table 5:** Top publishing journals

Journal	Number of Documents	Number of Citations
European Heart Journal	22	65
Circulation	19	706
BMC Cardiovascular Disorders	12	72
Journal of the American College of Cardiology	12	248
Journal of Interventional Cardiac Electrophysiology	11	90
Europace	10	630
Journal of Cardiovascular Electrophysiology	10	342
Circulation Journal	9	129
Frontiers in Cardiovascular Medicine	8	51
International Journal of Cardiology	8	116
Echocardiography: A Journal of Cardiovascular Ultrasound and Allied	6	58
PACE: Pacing and Clinical Electrophysiology	6	164
Clinical Cardiology	5	28
Heart Rhythm	5	392
Journal of Cardiothoracic Surgery	5	1
PLOS ONE	5	62
American Journal of Translational Research	4	30
Annals of Noninvasive Electrocardiology	4	23
Cardiovascular Drugs and Therapy	4	20
Journal of Arrhythmia	4	13
American Journal of Cardiology	3	178
Cardiology	3	8
Clinical Interventions in Aging	3	10
Heart	3	0
Heart and Vessels	3	33
Journal of Geriatric Cardiology	3	8
Medical Science Monitor	3	30
Swiss Medical Weekly	3	2

STEM (Science, Technology, Engineering, and Mathematics)

For the 359 institutions that have published at least three articles, as shown in Table 3, there are academic and corporate records from 182 countries, which include 240,078 documents (21.22 million citations and 258,820 total link strength) published as of October 30, 2023.

The most productive university was Capital Medical University, with the highest number of documents and link strength in the global research network at the university level. The first position was followed by Soochow University, Dalian Medical University, and Nanjing Medical University. These institutions exemplify China's dominance in research volume and connectivity. The top two data producers among the two relevant nations are from South Korea (Korea University) and Japan (University of Tsukuba), where Korea University contributed six documents and received 113 citations, whereas the University of Tsukuba produced far fewer documents, yet still received 328 citations. In Table 3, Japanese institutions (Sakurabashi Watanabe Hospital, Kyoto University) contribute to the global research network with a few citations and a high citation link strength.

More than half of the top 10 organizations are China-based, as evidenced by the predominant value of national publications and total link strength in Table 3, with other contributors to research being from Japan, South Korea, the Netherlands, and Belgium. For research collaboration done globally, the total link strength across institutions indicates the number of publications as well as international connections in research, and China leads the world in both. Moderate citation counts and link strengths are exhibited by institutions such as Leiden University, Université libre de Bruxelles, and Isala Klinieken.

Research impact and affiliations in Figure 4 (using VOSviewer) demonstrate intervals of respective connections based on co-authorship and citation relationships. A central cluster of Chinese institutions, including Capital Medical University and Soochow University, is evident, characterized by both high publication volumes and interconnectivity within the global research network. Institutions in Japan, South Korea, and the Netherlands are in more marginal positions, indicating somewhat limited yet significant international collaboration. China is a clear focal point for the network.

Keyword Analysis

**Figure 5:**
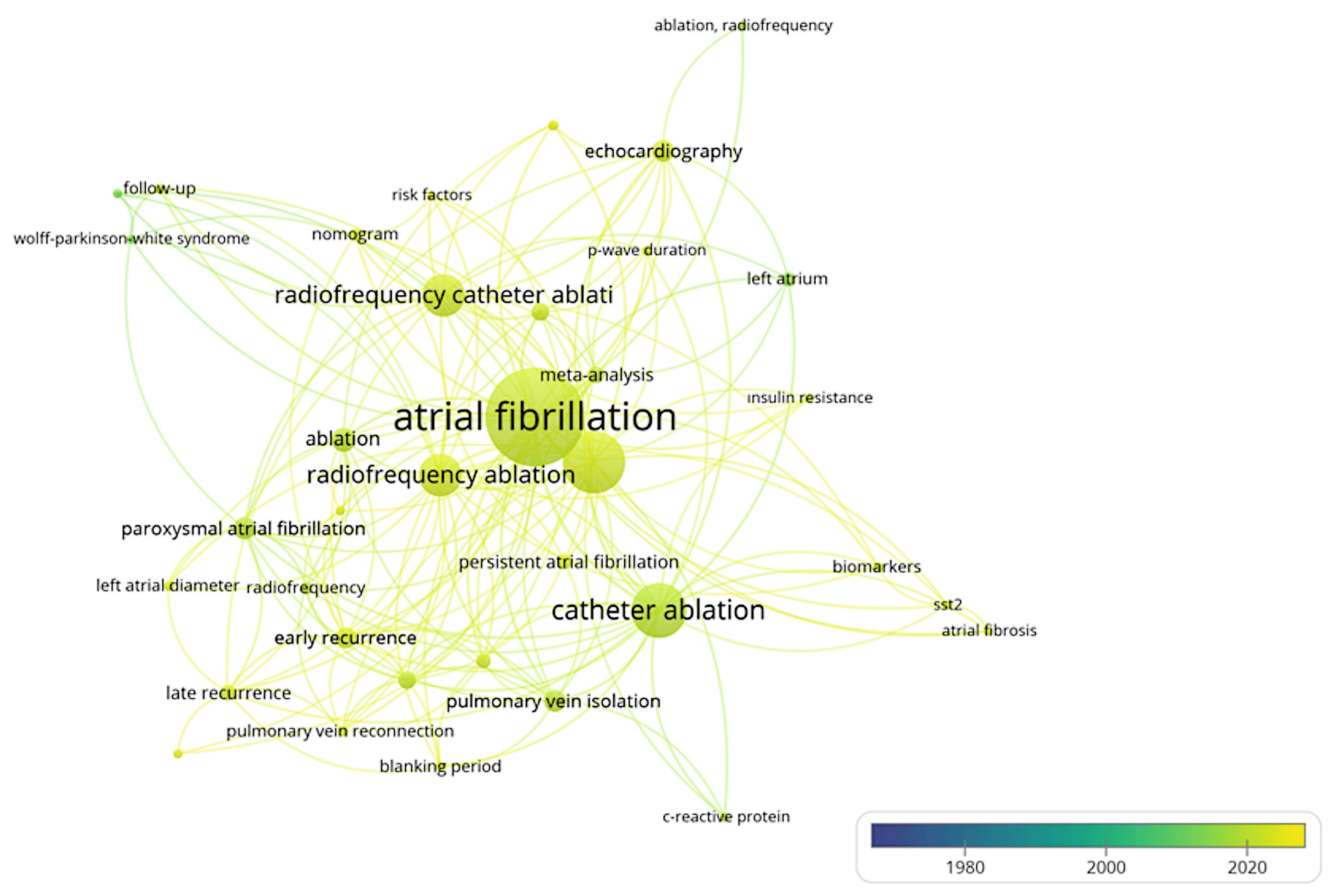
Keyword analysis in overlay visualization The color gradient indicates keyword frequency or prominence over time. The node size reflects the frequency of keyword occurrence. This overlay map visualizes trending topics in the research field. Hotter colors (red) may highlight emerging keywords, while node size shows their overall prevalence. Image Credits: Mehmet Semih Belpinar

The study utilized a total of 339 keywords, with 36 of them appearing at least three times. The trend keywords around recurrent AF post-radio-frequency (AF post-RFA) ablation are summarized in Table 6, which indicates the main research topics and hotspots. The most common keywords are "Ablation", followed by "Ablation, Radiofrequency" and "Accessory Pathway". Additional search terms were "Atrial Fibrillation" and "Atrial Fibrillation Recurrence", clearly centering the work on the topic of recurrence. "Atrial Fibrosis" and "C-Reactive Protein" are also clinical factors that shed light on the role of underlying cardiac conditions and inflammation in recurrence. Words such as "Cryoballoon Ablation" and "Catheter Ablation" indicate its significance by the different types of ablation. Patient characteristics responsible for outcome differences, like "Heart Failure" and "Left Atrial Diameter". Keyword analysis using the bibliometric tool VOSviewer, as shown in the visualization above (Figure 5), is created, which analyzes keywords for their importance over the years, outlining their significance and connections from 1990 to 2025. The richer the color and the larger the word, the more frequently those keywords have been trending in the literature recently.

**Table 6:** Keyword analysis "Radiofrequency Catheter Ablation" and "Radiofrequency Catheter Ablation (RFCA)", even though they are the same subject, can give different article results because of the writer's choice of names. It is included to maintain data integrity, and the mathematical weight is different. SST2: Soluble Suppression of Tumorigenicity 2

Keyword	Number of Occurrences	Total Link Strength
Ablation	146	289
Ablation, Radiofrequency	69	156
Accessory Pathway	56	119
Atrial Fibrillation	37	87
Atrial Fibrillation Recurrence	36	70
Atrial Fibrosis	14	26
Biomarkers	12	31
Blanking Period	12	24
C-Reactive Protein	12	32
Catheter Ablation	11	30
Cryoballoon	9	19
Cryoballoon Ablation	9	25
Early Recurrence	8	22
Echocardiography	7	19
Follow-Up	7	13
Heart Failure	6	17
Late Recurrence	6	14
Left Atrial Diameter	5	14
Left Atrium	5	13
Meta-Analysis	4	9
Nomogram	4	14
P-Wave Duration	4	13
Paroxysmal Atrial Fibrillation	4	7
Persistent Atrial Fibrillation	4	12
Prediction Model	4	13
Pulmonary Vein Isolation	3	4
Pulmonary Vein Reconnection	3	10
Radiofrequency	3	10
Radiofrequency Ablation	3	8
Radiofrequency Catheter Ablation	3	8
Radiofrequency Catheter Ablation (RFCA)	3	9
Recurrence	3	3
Risk Factors	3	9
SST2	3	10
Wolff-Parkinson-White Syndrome	3	9
Insulin Resistance	3	8

Discussion

This bibliometric study reveals a growing global interest in the topic of recurrent AF after radiofrequency ablation, accompanied by a significant increase in publications, particularly in 2023 and 2024. These trends can be attributed to advancements in health technology and its increased clinical application, as well as the growing awareness of the burden of recurrent AF. There is an inherent limitation of bibliometric analysis, as it cannot accurately reflect research quality and scientific innovation, and is also influenced by database coverage [7-9]. This study presents the total number of publications in the entire literature and the number determined as a result of the search according to the inclusion criteria in the WOS database.

The comparative lack of AF studies in China relative to Europe and the United States was recently described in a review of the recent literature. The prevalence of AF in community-based studies ranged from 0.49% to 8.8%, while in hospital-based studies, the frequency varied from 4.4% to 35.7%. This study highlights the need for more high-quality research and improvements in population-based follow-up of antithrombotic therapy in China due to China's large population and high prevalence of untreated patients with AF [26], which poses a potential burden on healthcare resources. The results of this study indicate that China's lead in research publications is largely due to its increasing prominence in this field of research, with major publications and a significant citation impact from institutions such as Capital Medical University and Soochow University. This may suggest that China is contributing to a greater output of research, while the USA has fewer articles, potentially indicating a higher impact through the creation of higher-quality works.

The findings of this study highlight the important role of international co-authorship, with China, Japan, and the USA representing the nucleus, providing a shared exchange of ideas, resources, and expertise to support scientific understanding. This bibliographic coupling between countries shows the worldwide study networks. Although some nations have lower link strengths due to limited collaboration or fewer publications, most nations are working together to solve recurring AF and patient outcome issues globally.

In this study, we analyze international research productivity and collaboration on the topic of AF. Against this backdrop, this novel underlines China's significance in this domain. China leads in research productivity and international collaborations, generating the most AF-related research. Nevertheless, current studies of AF screening in China are hampered by challenges related to technology and research methods. This emphasizes the urgent need for advanced strategies for the early diagnosis of AF and for the prevention of its complications. Interestingly, this comes shortly after other organizations based on larger populations, such as the US Prevention Services Task Force (USPSTF) and the UK National Screening Committee, still do not back mass ECG screening for AF. However, in recent years, technological advances have laid the foundation for novel approaches to AF screening. Using wearables (e.g., smartwatches like the Apple Watch in the Apple Heart Study), AF detection has advanced rapidly. In this study, 0.52% of participants were found to have AF over a three-month follow-up period [27,28]. China's productivity in AF research and the potential for global cooperation may facilitate the timely detection and prevention of complications such as stroke. Even if China is a leader in research, it also requires developing innovative solutions even in the worst of times. Novel approaches and technologies for AF screening need additional evaluation, particularly in larger populations, to identify more effective solutions to AF-related public health issues.

The current frontiers and hotspots of this discipline may be summarized based on the most cited publications and important keywords [29]. The keyword analysis indicates the main points of the research in this study. The treatment method is strongly centered on ablative studies, as "ablative" is the most frequently used keyword in these studies. It addresses the challenges patients face after treatment and the recurrence of AF. Clinical factors, exemplified by atrial fibrosis, as well as C-reactive protein, a marker of inflammation, emphasize inflammation as a potential factor in recurrence. Additionally, there are interventional techniques, along with their associated risks, including procedures such as cryoballoon and catheter ablation. Keywords have been interrelated to each other, with less emphasized terms such as "left atrial diameter", which were used more increasingly over time. It is increasingly recognized that inflammatory mediators can be produced in response to the immune microenvironment, substantially affecting treatment outcomes. One size does not fit all in terms of treatment interventions, and various factors also confound recurrence and recovery for individual patients.

To the best of our knowledge, this is the first bibliometric analysis of research on related recurrent AF after radiofrequency ablation, and this study offers an overview to help researchers better understand the hotspots, status, and trends in recurrent AF after radiofrequency ablation research. To the best of our knowledge, this bibliometric analysis is the first to present insights into the global research of recurrent AF after radiofrequency ablation. It shows the rise in publications, the leading countries and institutions, and the impact of key studies. However, a significant gap still exists between scientific knowledge and clinical practice.

Limitations

This study shows the total number of publications in the entire literature and the number determined as a result of the search according to the inclusion criteria in the WOS database. There are some limitations to the bibliometric analysis of AF recurrence after radiofrequency ablation, including the exclusion of non-English articles, using only the WOS database, the study being cross-sectional, data being collected on just one day, and the exclusion of irrelevant and non-English articles from journals. The bibliometric nature of the study may have introduced language bias due to the selection of English-language publications only. The database selection does not include all journals or publications, especially those that pertain to new or niche research areas. Additionally, even if WOS is the best source for SCI-E grade publications, citations do not always reflect the study's relevance and quality, and the study's specialization may dilute the interpretation of its general clinical implications.

## Conclusions

This study gives researchers a perspective on the layout of the field. Publications on new studies for recurrent AF after radiofrequency ablation have dramatically increased, particularly in 2023 and 2024. China is the top contributor, and the United States ranks highest in citation impact. The document analysis identified several key factors that contribute to the recurrence of AF, highlighting the implications of anatomical and clinical features on modifying the case's outcome. Eight countries were responsible for the majority of the citations, with China dominating the citations, followed by Japan and the USA, with France and the UK trailing further behind. Chinese organizations, such as Soochow University and Capital Medical University, exhibit strong global connections. South Korea, Italy, and Belgium had a relatively high proportion of collaborative articles based on their total share of publications. Additionally, cardiovascular journals such as Circulation and the European Heart Journal published many of the influential studies. With all the inferences, future work must be multidisciplinary and international, targeting both scientific and clinical challenges, to enable the development of more effective treatment approaches.
